# BCG-induced pneumonitis with lymphocytic pleurisy in the absence of elevated KL-6

**DOI:** 10.1186/1471-2466-14-35

**Published:** 2014-03-05

**Authors:** Makoto Tobiume, Tsutomu Shinohara, Takahira Kuno, Shinji Mukai, Keishi Naruse, Nobuo Hatakeyama, Fumitaka Ogushi

**Affiliations:** 1 Division of Pulmonary Medicine, National Hospital Organization National Kochi Hospital, 1-2-25 Asakuranishimachi, Kochi 780-8077, Japan; 2 Department of Clinical Investigation, National Hospital Organization National Kochi Hospital, 1-2-25 Asakuranishimachi, Kochi 780-8077, Japan; 3 Division of Urology, National Hospital Organization National Kochi Hospital, 1-2-25 Asakuranishimachi, Kochi 780-8077, Japan; 4 Division of Clinical Laboratory, National Hospital Organization National Kochi Hospital, 1-2-25 Asakuranishimachi, Kochi 780-8077, Japan; 5 Division of Pathology, National Hospital Organization National Kochi Hospital, 1-2-25 Asakuranishimachi, Kochi 780-8077, Japan

**Keywords:** BCG immunotherapy, Hypersensitivity pneumonitis, Bronchoalveolar lavage

## Abstract

**Background:**

Pneumonitis is a rare complication of bacillus Calmette-Guerin (BCG) immunotherapy seen in patients with urothelial cancer following the repeated administration of BCG. However, no case of BCG-induced pleurisy has been reported.

**Case presentation:**

We here report the first case of pneumonitis with lymphocytic pleurisy following bacillus Calmette-Guerin (BCG) immunotherapy. Although marked T helper cell alveolitis was found by bronchoalveolar lavage and transbronchial biopsies, no acid-fast bacillus could be identified in recovered BALF or pleural effusion. The lymphocyte stimulation test of BCG was strongly positive. However, levels of serum and bronchoalveolar lavage fluid KL-6, a useful marker for hypersensitivity pneumonitis (HP), were within normal ranges.

**Conclusion:**

We speculate that the pathogenesis of our case may be a hypersensitive reaction to the proteic component of BCG entering the lung and pleural space, which is different from the etiology of the common type of HP.

## Background

The intravesical or intrapelvic administration of bacillus Calmette-Guerin (BCG) has been proven effective against urothelial cancer. Compared to commonly induced granulomatous inflammatory changes in the bladder, pneumonitis is a rare complication of this immunotherapy that is seen in less than 0.7% patients following the repeated administration of BCG [1]. Because mycobacteria are not detected in these patients, a hypersensitivity reaction rather than a disseminated BCG infection is suspected in the pathogenesis of this disorder [2]. Epithelioid noncaseating granulomas of the lung have been identified in several cases [3]. The frequency of complications associated with BCG immunotherapy was shown to be significantly higher in patients with prior tuberculosis than in patients without a history of tubercular illness, which provides additional support for this hypothesis [4].

KL-6 is a mucin-like high-molecular-weight glycoprotein expressed on type 2 alveolar pneumocytes and bronchiolar epithelial cells, and was shown to be a useful serum or bronchoalveolar lavage fluid (BALF) marker for interstitial lung diseases including idiopathic interstitial pneumonia, pneumonitis related to collagen disease, and hypersensitivity pneumonitis (HP) caused by the repeated inhalation of organic antigens [5,6].

Iatrogenic pleurisy is a rare disease that is mainly induced by the systemic administration of certain agents and is characterized by eosinophilic pleural effusion. However, drug-induced lymphocytic non-eosinophilic pleurisies are rare [7]. In addition, to the best of our knowledge, there have been no reports of HP with pleural effusion.

We here report a case of interstitial pneumonia with lymphocytic pleurisy in the absence of elevated serum/BALF KL-6 following intrapelvic BCG immunotherapy.

## Case presentation

An 86-year-old female with a history of left renal cell carcinoma was diagnosed with right ureter cancer, and intrapelvic BCG therapy using a single-J stent was started with the mycobacterium bovis Connaught strain at 81 mg once a week. Prophylactic treatment of the disseminated BCG infection with a combination of isoniazid and rifampicin was started at the same time. The patient exhibited a continuous fever followed by dyspnea 6 days after the third BCG treatment.

**Figure 1:**
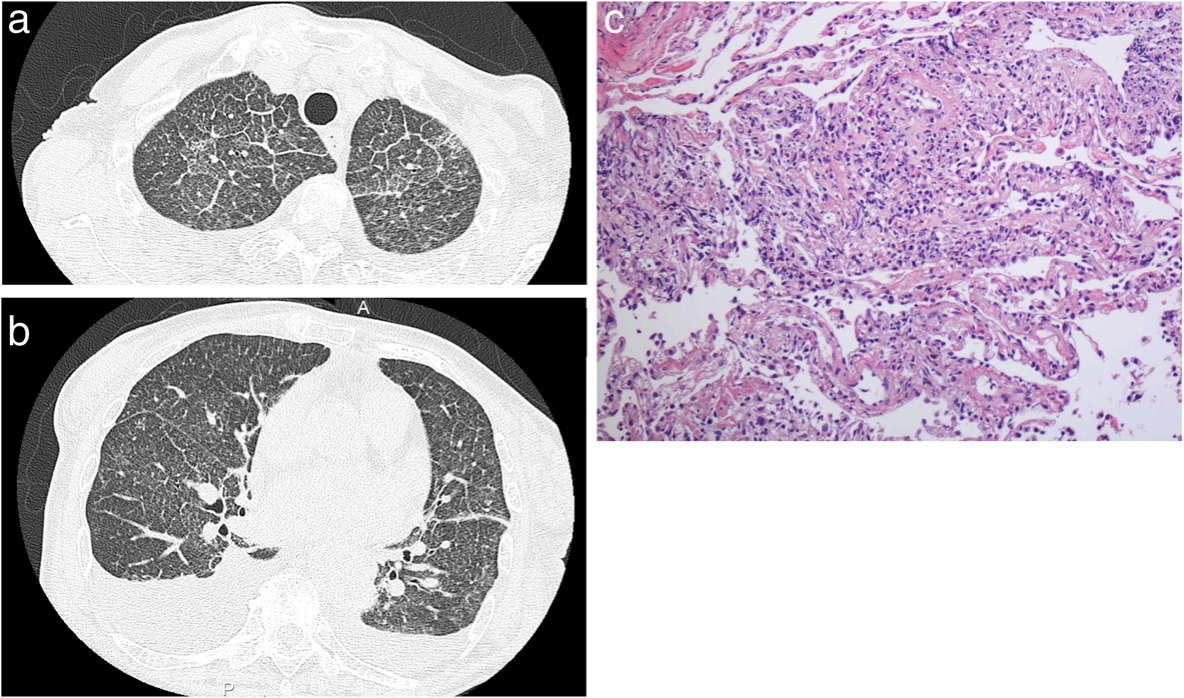
**HRCT of the a) upper and b) lower lung field, and c) histology of transbronchial lung biopy.** Ground-glass opacity, interlobular septal thickening, diffuse micronodular shadows, and bilateral pleural effusion are visible **(a, b)**. Alveolar septal thickening and lymphocyte infiltration are shown **(c)**.

A physical examination indicated fine inspiratory crackles in both lungs with no heart murmur. Laboratory data were as follows: white blood cell count, 6590/μl (neutrophils, 80.7%; lymphocytes, 11.8%; eosinophils, 2.1%; monocytes, 4.6%; basophils, 0.8%); hemoglobin, 13.2 g/dl; platelet count, 24.7 x 10^4^/μl; C-reactive protein, 6.07 mg/dl; serum KL-6, 171 U/ml. Severe hypoxemia (SpO2 86%) was noted. Chest X-ray revealed bilateral diffuse reticulonodular infiltrates and was suggestive of pleural effusion. High-resolution computed tomography (HRCT) showed ground-glass opacity, interlobular septal thickening, diffuse micronodular shadows, and bilateral pleural effusion (Figure 1a,b).

Bronchoalveolar lavage (BAL) (recovery rate; 84/150) revealed high total cellularity (70 × 10^4^ cells/ml), consisting of 84% lymphocytes, 11% neutrophils, and 5% eosinophils. Immunophenotyping showed that lymphocytes were 67% CD4+ cells and 12% CD8+ cells, with a CD4+/CD8+ ratio of 5.5. Direct microscopy, culture, and polymerase chain reaction of the BALF remained negative for pathogens including mycobacteria. KL-6 in the BALF was 217 U/ml (cutoff level was 340 U/ml [6]). The concentration of interleukin (IL)-8 in the BALF was 76.7 pg/ml (< 15 pg/ml [8]). IL-1β, IL-10, IL-12, interferon gamma (IFN-γ), tumor necrosis factor alpha (TNF-α), and monocyte chemotactic protein-1 (MCP-1) were under the detection limits (< 10, < 2, < 7.8, < 1.56, < 0.55 and < 62.5 pg/ml, respectively). Transbronchial biopsies of the right lung (rS3 and rS8) revealed alveolar septal thickening and lymphocyte infiltration with no evidence of mycobacteria (Figure 1c). A typical epithelioid noncaseating granuloma was not found in the tissue. Although a case of acute eosinophilic pneumonia associated with intravesical BCG therapy was reported previously [9], clear eosinophil infiltration was also not observed. An examination of the pleural effusion revealed lymphocytic pleurisy; 68% lymphocytes, 17% neutrophils, 14% macrophages, and 1% eosinophils. Adenosine deaminase (ADA) in the pleural effusion was 18.8 U/l (5.0-20.0). Pleural needle biopsy was not performed due to the insufficient buildup of effusion. The peripheral blood lymphocyte stimulation test (LST) of BCG was strongly positive (stimulation index 345%).

Immunotherapy with BCG was discontinued because complications appeared to be due to a hypersensitivity reaction to BCG, and methylprednisolone pulse therapy (500 mg × 3 days) was started followed by a reduction in the dose of prednisolone to 25 mg daily. Clinical symptoms, hypoxemia, and abnormal findings on chest X-ray improved after 1 month of this treatment. Prednisolone was tapered to complete withdrawal in a 2-month period.

## Discussion

In this case, pulmonary complications arose despite prophylactic treatment with a combination of isoniazid and rifampicin, and no acid-fast bacillus could be identified in recovered BALF or pleural effusion. Moreover, transbronchial lung biopsy revealed lymphocytic inflammation without the detection of mycobacterium. The patient reacted well to steroid therapy, which also confirmed that a disseminated BCG infection was unlikely.

In contrast to the decreased CD4+/CD8+ ratio reported in typical HP patients, marked T helper cell alveolitis was found by BAL, as reported in previous cases [2]. Since CD4+ alveolar lymphocytosis in the healthy zone of patients with localized pulmonary tuberculosis was reported previously [10], an increased CD4+/CD8+ ratio in BAL may imply intense immunosensitivity toward BCG antigens. Although IFN-γ, IL-12, and TNF-α, which were shown to be involved in the pathogenesis of HP in animal models and *in vitro* experiments [11,12], did not reach detectable values in the BALF of our patient, the high level of IL-8, which is a chemoattractant for T lymphocytes and neutrophils, was consistent with that in patients with HP [8].

LST is an examination that examines the sensitivity of a suspected material by measuring the proliferation of T cells to the material *in vitro,* from which one can conclude a previous *in vivo* reaction due to sensitization [13]. Since the LST of BCG was positive in our case, the hematogenous spread of the proteic component of BCG during a traumatic third endoscopic treatment has favored a hypersensitive reaction in a previously sensitized patient.

Patients with pulmonary tuberculosis or tuberculous pleurisy have significantly higher levels of serum KL-6 than those of healthy controls. However, the mean values of these tubercular illnesses were reported to be less than 500 U/ml [14]. In contrast, circulating KL-6 levels were shown to be markedly increased in particular types of drug-induced lung injury such as diffuse alveolar damage and chronic interstitial pneumonia patterns [15], and an elevated serum KL-6 level is one characteristic of active HP (1,508 ± 647 U/ml [5]). In addition, Kohno et al. reported that a high level of KL-6 in the BALF was observed in 100% (8 of 8) of patients with HP [6]. Another study indicated that serum KL-6 levels did not increase in drug-induced pneumonitis with a HP pattern (diffuse ground-glass opacities without fibrosis) [15]. Although KL-6 levels in patients with interstitial pneumonia secondary to intravesical BCG immunotherapy have not been evaluated previously, serum and BALF KL-6 levels in our patient were within normal ranges. The difference observed in KL-6 levels suggests that pulmonary epithelial cell injury caused by BCG-induced pneumonitis may not be as prominent as that of the common type of HP.

Caramori *et al.* recently reviewed the community-acquired pulmonary complications of intravesical BCG immunotherapy in 15 patients, including their own patient. Chest imaging of these cases revealed a military pattern (n = 12), bilateral pulmonary opacities (n = 2), and reticulonodular pattern (n = 1). However, the pleural effusion was not included [16]. Although BCG-induced parenchymal infiltration with a modest homolateral pleural reaction was observed in a patient with COPD and a history of previous tuberculosis in one case report, the presence of pleural effusion and details of the “pleural reaction” were not described [17].

The main disease that shows lymphocytic pleural effusion is tuberculous pleurisy. The pathogenesis of primary tuberculous pleurisy is a hypersensitivity immunogenic reaction to a few mycobacterial antigens entering the pleural space rather than direct tissue destruction by uncontrolled mycobacterial proliferation [18]. An injection of BCG into the pleural space of animals previously sensitized by intradermal BCG can induce experimental tuberculous pleurisy [19]. These findings suggest that the hypersensitivity reaction to BCG antigens may be the mechanism of lymphocytic pleurisy in our patient.

The number of macrophages was larger in the pleural effusion than in the BALF in this case. Although the role of macrophages in tuberculous pleurisy remains unclear, stimulated human pleural mesothelial cells were shown to produce monocyte chemotactic protein-1 (MCP-1), which is known to be elevated in tuberculous pleural fluid [20,21]. Interactions between macrophages and lymphocytes may have been present in the pleural space of our patient.

We could not analyze the antigen-antibody reaction in our patient. However, the presence of serum-specific IgG to BCG was reported previously in a patient with BCG-induced pneumonitis [22]. In addition, the level of ADA, which is related to the proliferation of T lymphocytes, in the pleural effusion of our patient was not higher than that in patients with typical tuberculous pleurisy. These findings suggest that the hypersensitivity reaction was induced not only by cell-mediated immunity, but also by humoral immunity in patients treated with BCG.

## Conclusion

This is the first case report of interstitial pneumonia with lymphocytic pleurisy following BCG immunotherapy. We suggest that the pathogenesis of our case may have been a hypersensitive reaction to the proteic component of BCG entering the lung and pleural space, which is different from the etiology of the common type of HP.

### Consent

Written informed consent was obtained from the patient for publication of this case report and any accompanying images. A copy of the written consent is available for review by the Editor-in-Chief of this journal.

## Abbreviations

BCG: Bacillus calmette-guerin; BALF: Bronchoalveolar lavage fluid; HP: Hypersensitivity pneumonitis; HRCT: High-resolution computed tomography; LST: Lymphocyte stimulation test.

## Competing interests

The authors declare that they have no competing interests.

## Authors’ contributions

MT drafted the initial manuscript. TS edited and submitted the manuscript. TK was involved in diagnosing and treating the patient. SM contributed to the collection of patient data. KN performed pathological studies. NH and FO were the attending physicians throughout the disease course. All authors read and approved the final manuscript.

## Pre-publication history

The pre-publication history for this paper can be accessed here:

http://www.biomedcentral.com/1471-2466/14/35/prepub
